# Canvassing

**Published:** 1901-12-14

**Authors:** 


### "CANVASSING."

No one can doubt that the intrusion into medical practice of the commercial methods adopted by medical aid societies has been productive of much harm to us as a profession. Not only have many practitioners been directly and seriously injured by loss of practice, but the general status and reputation of the profession has been dragged down by the acquiescence of men of apparently good professional standing in arrangements which involve the imperfect performance of their professional duties, and often in methods of advertising which are in every way to be deprecated. We can fully believe, then, that a very large number of medical men will hail with pleasure the announcement that the General Medical Council have plucked up courage, and have summoned before them the medical officer of one of these societies to answer a charge of being guilty of "infamous conduct in a professional respect" in that he has held an appointment in a society which "systematically practised canvassing for the purpose of procuring patients," and that he has acquiesced or approved of such canvassing. As a professional pronouncement against canvassing—if it may be taken as such—this action is to be heartily welcomed. As a display of worldly wisdom it is not of much account. No doubt the Council, knowing that they have been entrusted with large powers, feel that they are responsible for the morals of the profession, and that they are participators in what they fail to punish. That is one side of the question. We cannot shut our eyes, however, to the fact that there is another side, and that the application of the term "infamous" to conduct which to the rest of the world must appear harmless, even if ignominious, and the infliction of the severe punishment of expulsion from the Registrar for such offences, are likely to be attended with inconvenience, and perhaps even with danger to our position as a profession. We have to remember that hitherto the penal cases brought before the Council have generally concerned offences the continuance of which is clearly against the public interest, and that in punishing these offences the Council have been carrying out the original aim and intention of the Acts, namely, the protection of the public against the false pretences of unqualified persons. The matter, however, assumes a somewhat different aspect when the Council begin, if we may so say, to create offences. It is evident that to advertise and to canvass for patients, however infamous they may seem to us, are by no means heinous crimes according to any code of morality current among the public; and although it is true that, as the law stands, it lies with the Council to decide what is "infamous" and what is not, we would urge that the exercise of their punitive powers against purely professional offenders should be undertaken with the greatest circumspection. More especially should this be the case when the alleged offences are not concerned with actual professional work, but with what one may call the commercial side of practice, such as modes of payment and methods of obtaining patients. It is a serious matter, a matter greatly to be regretted, that medical men should be found willing to advance their interests by canvassing and advertising. But it would be a still more serious matter were the medical profession, or the public bodies by whom it is represented, to be found willing to strain an Act of Parliament passed in the interest of the public (for an Act must be read with its preamble) and to make use of it for the promotion of what must by those outside be regarded as the trade interests of the profession—and this is what the General Medical Council are on the verge of doing.

Dissatisfaction with the present legal status of the medical profession is the dominant note wherever medical reformers meet together, and many people are hoping great things from a reformed Medical Act. All hope of obtaining useful reform will, however, vanish if we manage to rouse any organised opposition; and although, of course, it is impossible to say what the medical aid societies will do, our general knowledge of human nature and parliamentary tactics leads us to expect that when next we ask for an amendment of the Medical Acts we shall have a very *mauvais quart d'heure.* The Medical Acts were passed for the protection of the public, not of ourselves, and if it should be shown or even made to appear that the administration of the Acts has been strained as against the public, the friendly societies would no doubt be delighted to combine to clip the claws of the General Medical Council.

